# Amoxicillin-Induced Hypersensitivity Versus Viral Exanthem in Epstein-Barr Virus Infection: A Paediatric Case Series

**DOI:** 10.7759/cureus.99781

**Published:** 2025-12-21

**Authors:** Mariana Viegas, Jacinta Mendes, Sónia Lemos, Estefânia Maia, Gina Rubino

**Affiliations:** 1 Pediatrics, Unidade Local de Saúde do Oeste, Caldas da Rainha, PRT; 2 Pediatric Allergy, Ambulatory Pediatric Department, Hospital Pediátrico de Coimbra, Unidade Local de Saúde de Coimbra, Coimbra, PRT

**Keywords:** amoxicillin, drug hypersensitivity, drug provocation tests, epstein-barr virus, exanthema

## Abstract

Epstein-Barr virus (EBV) infection is often associated with skin rashes following aminopenicillin treatment, such as amoxicillin, typically considered a benign and transient reaction. However, distinguishing between non-allergic exanthems and true hypersensitivity remains a clinical challenge, especially in children and adolescents.

This case series reports three cases of adolescents who developed generalized exanthematous eruptions following the administration of amoxicillin during acute EBV infection, confirmed by serological testing. All patients underwent standardized allergy evaluation through drug provocation testing at least ten weeks after rash resolution, which confirmed true delayed hypersensitivity to amoxicillin.

These three pediatric cases reinforce the need for heightened clinical vigilance when a skin rash occurs during EBV infection treated with amoxicillin. While such rashes are often attributed to transient viral-drug interactions, our findings highlight the importance of considering the possibility of true amoxicillin hypersensitivity. Careful evaluation, including comprehensive allergy work-up when appropriate, is essential to accurately identify a true aminopenicillin allergy and thereby reduce the risk of future severe hypersensitivity reactions.

## Introduction

Epstein-Barr virus (EBV) is a gammaherpesvirus commonly associated with infectious mononucleosis (IM), but it has also been linked to both malignant conditions, such as Burkitt lymphoma and Hodgkin lymphoma, and non-malignant infection-related diseases, including Gianotti-Crosti syndrome, erythema multiforme, and acute genital ulcers [1,2].

The seroprevalence of EBV in the general population goes up to 80% in children and 90-95% in adults, particularly in developed countries [1,3].

While EBV is mostly transmitted through saliva, it can also be spread via breastfeeding, other bodily fluids, and through the transplantation of organs from EBV-positive donors [1,4]. It has a latency period of four to eight weeks [5]. Around 50% of children under the age of six with a primary symptomatic EBV infection develop IM, a self-limiting lymphoproliferative condition that typically has a favorable prognosis [6].

IM typically manifests through a triad of symptoms (fever, pharyngitis and enlarged lymph nodes); however in a number of cases, it can present with hepatosplenomegaly, fatigue, and headache [5-7]. A rash is a common dermatological manifestation of EBV-induced IM and may appear in 4-13% of patients even in the absence of drug intake [8]. The characteristic rash usually appears as a morbilliform eruption primarily affecting the trunk, but it may also present as macular, petechial, scarlatiniform, or urticarial [9].

After antibiotics were initially evaluated for the treatment of IM in the 1940s and 1950s, an increased incidence of skin rashes in these patients was observed, causing the presence of a rash associated with antibiotic therapy to become, to a certain degree, pathognomonic for IM [8,9].

Cutaneous eruptions during antibiotic treatment in the presence of viral infections are frequently observed. However, the immunopathogenic mechanisms linking viral modulation to drug hypersensitivity remain unclear, complicating the differentiation between true drug allergies and transient virus-drug interaction reactions. [10,11].

This article aims to present three clinical cases of adolescents who developed an amoxicillin-induced exanthem in the context of serological evidence of recent EBV infection, and who were subsequently found to have a confirmed true hypersensitivity to amoxicillin following allergy workup through drug provocation testing. 

We conducted a retrospective review of medical records from patients diagnosed with amoxicillin hypersensitivity who developed cutaneous eruptions during amoxicillin treatment for IM. All cases were followed at the Pediatric Allergy Consult of Coimbra's Paediatric Hospital.

Data collected included clinical presentation, details of the diagnostic work-up, management approaches, and follow-up outcomes. Written informed consent was obtained from the parents or legal guardians of all patients included in the study.

This article was previously presented as a meeting abstract at the 2025 National Paediatric Congress of the Portuguese Paediatric Society.

## Case presentation

Case 1

**Figure 1:**
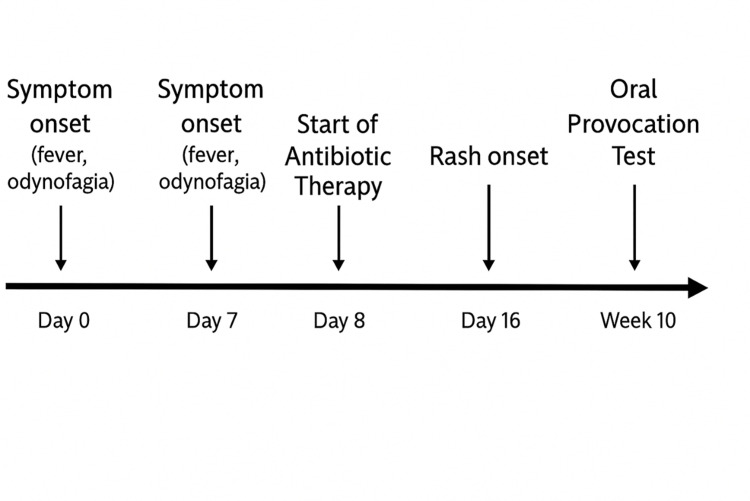
Case 1: Timeline of clinical events and rash development

A 16-year-old male patient, with a known history of allergic rhinitis, presented to the Pediatric Emergency Department (ED) with a 12-hour history of fever and odynophagia. Physical examination revealed enlarged tonsils with hyperemia and purulent exudate, along with a diffuse, towel-like erythematous rash over the trunk. A rapid streptococcal antigen test performed on an oropharyngeal swab was negative. Laboratory investigation showed an elevated white blood cell (WBC) count (20,000/µL) (reference range: 4,500-11,000) with neutrophilia (16,000/µL) (reference range: 1,800-7,700), and elevated C-reactive protein (CRP) at 9 mg/dL (reference range: < 0.5) with normal procalcitonine (0.07 ng/mL) (reference range: < 0.5). EBV and cytomegalovirus serologies were negative. The patient was evaluated by the otorhinolaryngology (ENT) team, who excluded local complications. He returned the following day for reassessment, with sustained apyrexia and clinical improvement. Seven days later, the patient returned to the ED with a 24-hour history of fever. Physical examination revealed bilaterally enlarged, erythematous tonsil. Additionally, a faint macular rash was observed on the trunk. A rapid streptococcal antigen test was positive, and antibiotic therapy with amoxicillin was initiated. On the ninth day of amoxicillin, the patient developed a rash (Figure 1).

**Figure 2:**
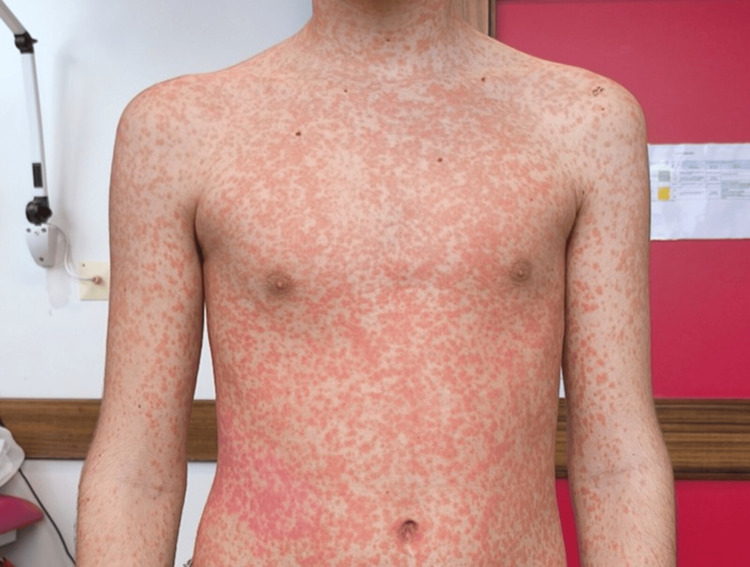
Exuberant morbilliform exanthem involving the chest and upper limbs

**Figure 3:**
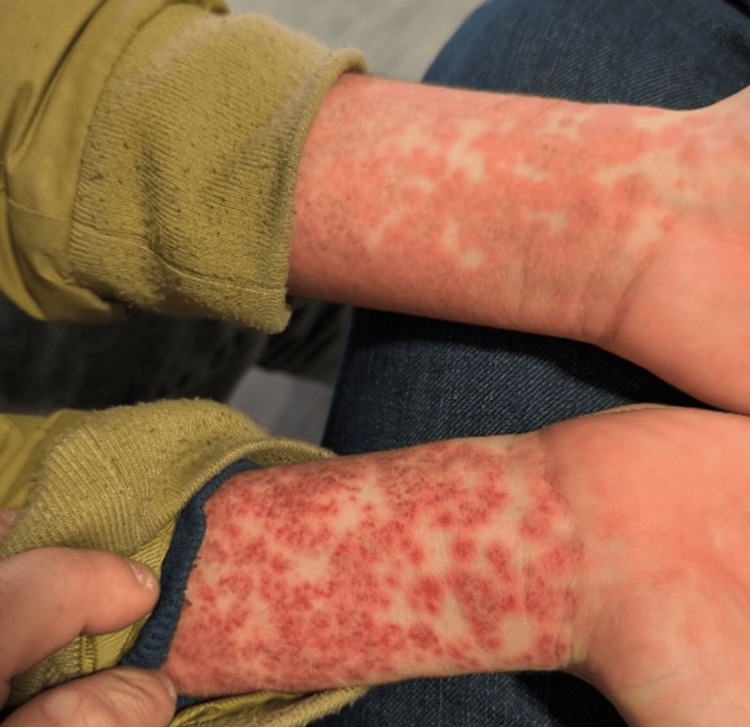
Exuberant morbilliform exanthem involving the wrists

He was afebrile since day two of amoxicillin. The patient had a prior history of amoxicillin use without any reported hypersensitivity reactions. On examination, an exuberant morbilliform exanthem was noted, involving the face, chest, back, and upper limbs. There was no involvement of the palms or soles (Figures 2 and 3).

The oropharynx showed marked erythema and tonsillar hypertrophy with prominent exudate. Additionally, bilateral palpable cervical lymphadenopathy was noted, with no evidence of hepatosplenomegaly. Serological testing suggested recent EBV infection with positive EBV-viral capsid antigen (VCA) immunoglobulin M (IgM) and immunoglobulin G (IgG) antibodies. The patient improved on antihistamine therapy. A possible virus-drug reaction was considered, and an allergology consultation was requested to rule out amoxicillin allergy.

An oral provocation test with amoxicillin was performed 10 weeks after the acute episode (Figure 1), with the development of a morbilliform rash on the first day of antibiotic administration, confirming hypersensitivity to amoxicillin.

Case 2

A previously healthy 15-year-old female patient, with no known history of allergic disease, presented to the ED with a seven-day history of fever and severe odynophagia. She had been empirically started on amoxicillin-clavulanate three days prior, without prior rapid streptococcal testing and no clinical improvement. 

**Figure 4:**
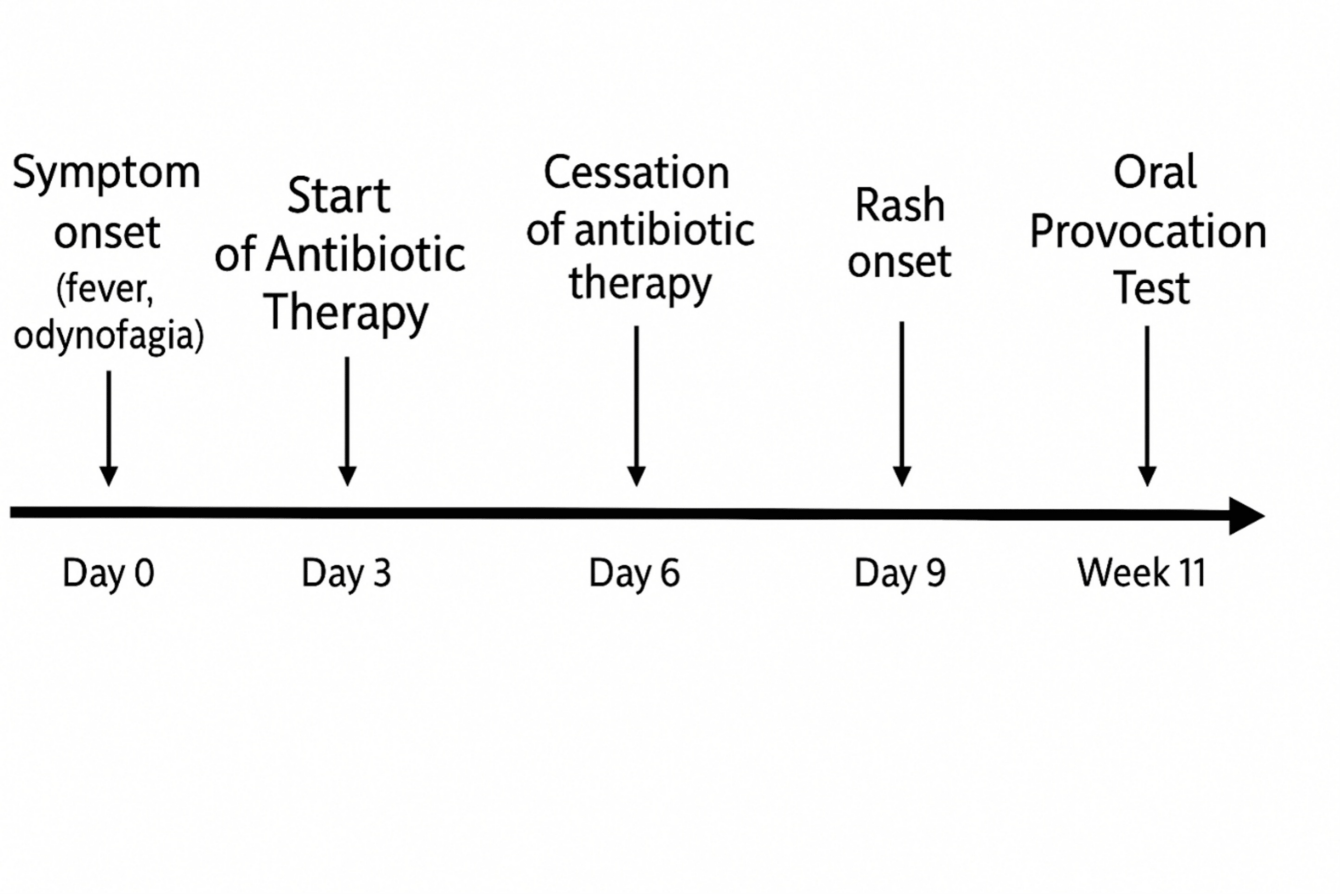
Case 2: Timeline of clinical events and rash development

**Figure 5:**
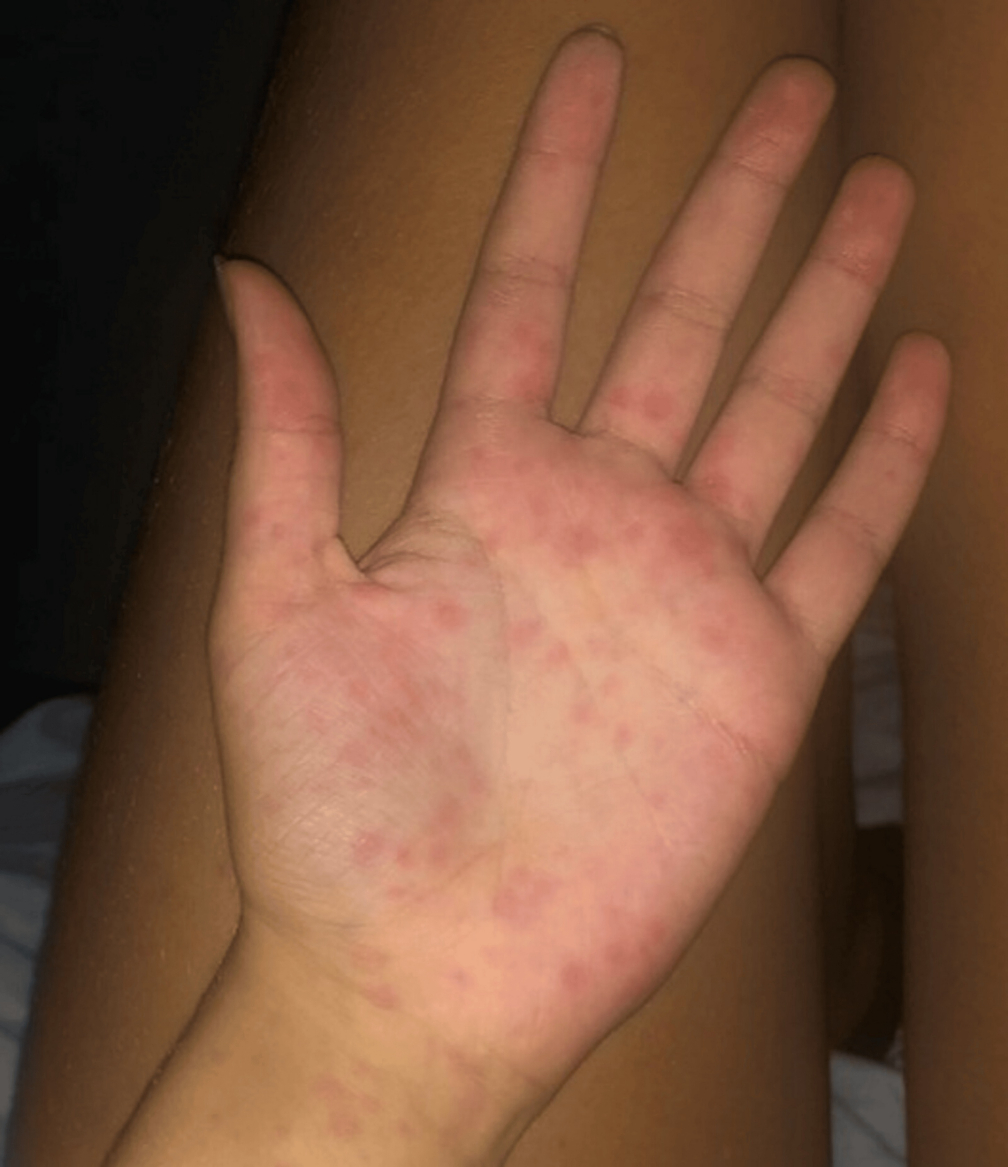
Micropapular rash with involvement of the palms

On examination, grade IV tonsillar hypertrophy with bilateral purulent exudate was observed, along with reactive bilateral anterior cervical lymphadenopathy. Laboratory results showed elevated liver enzyme levels, with aspartate aminotransferase (AST) at 81 U/L (reference range: < 35) and alanine aminotransferase (ALT) at 92 U/L (reference range: < 45), CRP of 2.55 mg/dL (reference range: < 0.5) and elevated WBC count at 18,200/uL (reference range: 4,500-11,000). The monospot test was positive. A diagnosis of IM was made, and amoxicillin-clavulanate was discontinued. The patient was readmitted to the ED five days later due to generalized pruritic rash that started on day 4 of antibiotic treatment (Figure 4). On examination, there was a micropapular rash on the face, trunk, and limbs, with involvement of the palms and soles (Figure 5). There was improvement of the rash following antihistamine therapy. The patient had no prior history of antibiotic use. An oral amoxicillin provocation test was conducted 11 weeks later (Figure 4), which was positive, as a morbilliform rash developed on the fourth day of antibiotic administration, confirming a hypersensitivity reaction to amoxicillin.

Case 3

**Figure 6:**
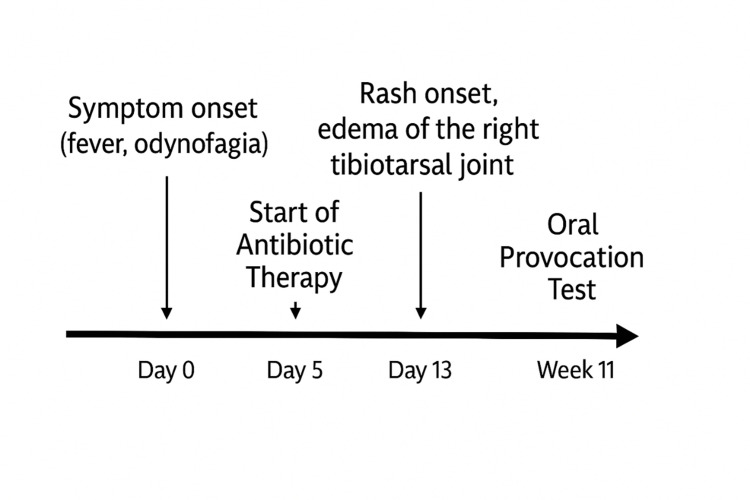
Case 3: Timeline of clinical events and rash development

A 12-year-old female patient, with no documented history of atopic or allergic disorders, was admitted to the ED on the ninth day of amoxicillin treatment due to a generalized rash, accompanied by arthralgia and mild edema of the right tibiotarsal joint . She had been on amoxicillin to treat symptoms of fever, odynophagia, abdominal pain, and nausea that lasted for six days (Figure 6), with a positive streptococcal antigen test result. The patient had a prior history of amoxicillin-clavulanate use without any reported hypersensitivity reactions. Physical examination revealed a generalized maculopapular rash affecting the face, trunk, and limbs, including the palms and soles. Additionally, bilateral cervical lymphadenopathy was noted, along with mild pain and swelling of the right tibiotarsal joint. Laboratory results showed a normal WBC count, CRP of 0.15 mg/dL (reference range: < 0.5) and elevated liver enzyme levels, with AST at 159 U/L (reference range: < 31) and ALT at 256 U/L (reference range: 9-25). Serological testing suggested recent EBV infection with positive EBV-VCA IgM and IgG antibodies. A probable diagnosis of serum sickness-like illness was made, with resolution of symptoms following the discontinuation of amoxicillin and antihistamine therapy. Normalization of liver enzyme levels was observed at the three-week follow-up.

An oral provocation test with amoxicillin was performed 11 weeks after the episode (Figure 6), resulting in a generalized urticarial rash and right wrist edema on the first day of treatment.

## Discussion

Skin rashes associated with EBV infection may arise either as a manifestation of the viral illness itself, typically four to six days after the onset of the disease, or as a reaction to concurrent antibiotic therapy, particularly with aminopenicillins, making the differential diagnosis challenging [12].

The concomitant use of aminopenicillins during EBV-associated IM was historically associated with a high incidence of skin rashes, reported in 80-100% of cases. However, more recent studies have demonstrated significantly lower rates [9].

A retrospective study conducted by Chovel-Sella et al. reported a rash incidence of 29.5% among patients with IM treated with amoxicillin, compared to 23% in those who did not receive antibiotic therapy [9]. These findings suggest a lower incidence of amoxicillin-induced rash in IM patients than that previously described in early studies, nonetheless, the incidence of confirmed drug hypersensitivity reactions among these patients was not established.

Traditionally regarded as benign and non-allergic reactions related to EBV-induced IM, amoxicillin-associated rashes in the context of classic IM have recently raised concerns, with emerging evidence suggesting that true drug hypersensitivity cannot be entirely ruled out [13,14]. This challenges the long-standing assumption that antibiotic-associated exanthems in the context of EBV infection are invariably non-allergic.

Rash pathogenesis likely involves immune responses to both EBV and amoxicillin. Although clinically similar, timing may aid differentiation: EBV-related rashes usually appear early and resolve with the illness, while amoxicillin-induced rashes emerge 3-10 days after treatment and are often more pronounced at presentation [15].

In our case series, Patient 2 developed a rash four days after discontinuation of amoxicillin-clavulante, whereas Patient 3 presented with a rash on the ninth day of ongoing amoxicillin therapy. In contrast, Patient 1 presented with a rash from the onset of illness, representing a significant diagnostic challenge in distinguishing between a typical EBV-associated eruption and a virus-drug interaction secondary to amoxicillin. The early presentation with acute tonsillitis and a persistent exanthem, along with a negative EBV IgM serology, was likely due to testing performed at an early stage of the disease, and positive rapid streptococcal antigen test, supported an initial diagnosis of scarlet fever and justified empirical treatment with amoxicillin. However, the subsequent clinical deterioration, with delayed EBV IgM seroconversion, suggested a concomitant EBV infection. To enhance the understanding of the temporal and clinical patterns observed, the key features of all three cases are summarized in Table 1. 

**Table 1:** Clinical comparison of the three cases ALT: Alanine aminotransferase; AST: Aspartate aminotransferase; CRP: C-reactive protein; EBV: Epstein-Barr virus; IgG: Immunoglobulin G; IgM: Immunoglobulin M; WBC: White blood cell

Variable	Case 1	Case 2	Case 3
Age	16 years	15 years	12 years
Day rash appeared	Day nine of amoxicillin	Day four after amoxicillin–clavulanate discontinuation	Day nine of amoxicillin
Morphology	Exuberant morbilliform exanthem involving face, chest, back, upper limbs; palms/soles spared	Generalized micropapular rash including palms/soles	Generalized maculopapular rash with palmoplantar involvement, edema of the right tibiotarsal joint
Laboratorial abnormalities	Leukocytosis, neutrophilia, ↑CRP, later positive rapid streptococcal antigen test and EBV IgM/IgG+	↑AST/ALT, ↑CRP, leukocytosis, positive monospot	↑AST/ALT, normal WBC count, normal CRP, EBV IgM/IgG+
Oral provocation timing	10 weeks after episode	11 weeks after episode	11 weeks after episode
Provocation result	Positive (morbilliform rash day 1)	Positive (morbilliform rash day 4)	Positive (urticaria + wrist edema day 1)

This evolution highlights the diagnostic overlap between streptococcal pharyngitis exanthem, EBV-associated viral exanthems, and drug-induced hypersensitivity reactions, particularly in patients exposed to aminopenicillins. While both drugs and infectious agents have been previously described as possible precipitants to the rash, the exact mechanism remains unknown [16].

Some authors propose that viral infections may transiently disrupt immune tolerance, enhancing drug reactivity through altered antigen presentation or immune dysregulation [15,16]. Nonetheless, in certain cases, these reactions may reflect a true sensitization to aminopenicillins, underscoring the importance of recognizing this entity and pursuing appropriate allergy workup and, therefore, identifying sensitized patients to potentially avoid severe reactions [13].

In a study by Misirlioglu et al., the authors sought to determine the prevalence of true antibiotic hypersensitivity among pediatric patients with IM confirmed by positive EBV IgM serology who developed a rash during antibiotic treatment. Within their cohort, 54.3% of children received antibiotics, and although the overall incidence of rash was comparable between antibiotic-treated and untreated patients, a subset of children (30% of those who underwent allergy evaluation) showed confirmed hypersensitivity to amoxicillin-clavulanate. Furthermore, five of the non-tested patients experienced recurrent rashes upon re-exposure to the suspected antibiotic, supporting the likelihood of true drug hypersensitivity in a subset of cases [17]. Allergy assessment in this study included skin testing for immediate-type reactions and/or oral provocation testing in cases of non-severe, delayed reactions, performed at least six weeks after clinical resolution [17]. Similarly, Renn et al. demonstrated that some young adults with EBV-related exanthems following amoxicillin exposure exhibited drug-specific sensitization, confirmed by positive skin tests and lymphocyte transformation tests (LTT) performed four-six weeks after rash resolution [14].

Our case series support these findings, suggesting that, while most exanthems in this context are self-limited and non-allergic, a proportion of patients may indeed develop true hypersensitivity reactions. All three adolescents developed maculopapular or morbilliform eruptions during amoxicillin treatment for EBV-confirmed IM and were later found to have confirmed true delayed- hypersensitivity to amoxicillin following standardized allergy workup. In Case 1, an oral provocation test was conducted 10 weeks after the reaction, while in Cases 2 and 3 it was performed 11 weeks post-reaction. Given that IgM antibodies to EBV VCA are typically detectable only within the first 2 to 3 months following symptom onset, the oral provocation tests in our cases were conducted at a time when IgM titers would be expected to have declined or become undetectable, thereby reducing the likelihood of false-positives due to a possible virus-drug interaction [18]. Taken together, these observations reinforce that while antibiotic-associated rashes in EBV infection are historically perceived as benign, non-allergic and self-limited, a subset of patients may develop true immunologically mediated hypersensitivity. Clinicians should maintain a high index of suspicion in such cases and pursue timely allergy testing, potentially avoiding adverse and severe reactions.

## Conclusions

This study highlights the complex interplay between EBV infection, the occurrence of exanthema during treatment with antibiotics, particularly amoxicillin, and suspected amoxicillin allergy. Historically, these cases have not been routinely referred for Allergy/Immunology evaluation. However, a thorough clinical assessment-including detailed history of prior amoxicillin exposure and any associated hypersensitivity reactions-is essential. Structured follow-up in an allergy clinic is recommended, with re-evaluation strategies that may include a repeated oral provocation test. This approach is critical to distinguish virus-induced reactions from true drug hypersensitivity, thereby reducing the risk of future adverse events. Importantly, the persistence of hypersensitivity following EBV infection remains uncertain, and although it may raise concern for adverse reactions with future antibiotic exposures, this possibility is theoretical and requires further investigation.
